# Acidification and nitrification inhibition of manure alters greenhouse gas emissions and nitrogen cycling in diverse agricultural soils

**DOI:** 10.1002/jeq2.70123

**Published:** 2025-12-14

**Authors:** Mitch D. Wodrich, Steven J. Hall, Xia Zhu‐Barker

**Affiliations:** ^1^ Department of Soil and Environmental Sciences University of Wisconsin‐Madison Madison Wisconsin USA; ^2^ Department of Plant and Agroecosystem Sciences University of Wisconsin‐Madison Madison Wisconsin USA

## Abstract

Manure amendments are widely used in agriculture to enhance crop productivity and maintain soil organic matter, but they also contribute to greenhouse gas emissions and reactive nitrogen (N) losses. Manure acidification and nitrification inhibitors (NIs) have been proposed as mitigation strategies, but their effectiveness likely varies with soil characteristics. To evaluate the influence of acidified and NI‐treated manure on N cycling across different soil types, we conducted a 28‐day laboratory incubation using soils collected from major agricultural regions in Wisconsin. We measured emissions of nitrous oxide (N_2_O), nitric oxide (NO), carbon dioxide (CO_2_), and methane (CH_4_), along with soil ammonium (NH_4_^+^) and nitrate (NO_3_^−^) dynamics. Acidified manure enhanced short‐term NH_4_^+^ retention in all soils but increased cumulative N_2_O emissions in the sandy soil compared to untreated manure, likely due to pH‐driven disruption of nitrification and incomplete denitrification. In contrast, the NI dicyandiamide (DCD) consistently suppressed nitrification early in the incubation, resulting in significantly lower N_2_O and NO emissions, often approaching levels observed in the no‐manure controls, particularly in the sandy soil. Manure amendments increased CO_2_ fluxes relative to the no‐manure controls, but acidified manure emitted less CO_2_ than DCD‐treated manure, likely due to temporary suppression of microbial respiration. CH_4_ emissions were minimal and largely unaffected by treatments. NIs offer consistent benefits in reducing N_2_O and NO losses, while acidification can increase these emissions in certain soil conditions, highlighting the importance of tailoring management practices to specific soil characteristics.

## Core Ideas

- Treating manure with a nitrification inhibitor significantly reduced soil N_2_O and NO emissions.
- Acidified manure increased N_2_O emissions in soils with low soil buffering capacity.
- Effect of acidification and nitrification inhibition of manure depends on soil characteristics.

## Plain Language Summary

Farmers use manure to improve soil health and crop growth, but it can also release greenhouse gases and nitrogen into the environment. Two strategies have been proposed to limit these losses: acidifying manure and adding nitrification inhibitors (NIs). In a 28‐day lab study using soils from across Wisconsin, we tested how these treatments affected greenhouse gas emissions and nitrogen cycling. Acidified manure helped soils hold more ammonium but increased nitrous oxide emissions in sandy soils, while the NI treatment (dicyandiamide) consistently reduced nitrous oxide and nitric oxide emissions, often to levels close to soils without manure. Manure additions increased carbon dioxide release, although acidified manure gave off less than NI‐treated manure, and methane emissions were very low across all treatments. Overall, NIs were effective in cutting nitrogen gas losses, while acidification sometimes increased emissions depending on soil type, showing that manure management needs to be tailored to specific soil types.

**Abbreviations**
AMO: ammonia monooxygenase
AOB: ammonia‐oxidizing bacteria
DCD: dicyandiamide
DI: deionized
DM: dry matter
DOC: dissolved organic carbon
GHG: greenhouse gas
M+A: manure + acid
M+NI: manure + nitrification inhibitor
NI: nitrification inhibitor
NOB: nitrite‐oxidizing bacteria
nosZ: nitrous oxide reductase
TC: total carbon
TN: total nitrogen
WHC: water holding capacity

## 1 INTRODUCTION

Manure application is a cornerstone of nutrient management in agriculture, particularly in livestock‐intensive regions like Wisconsin, where approximately 1.27 million dairy cows generate tens of millions of tonnes of manure per year, providing a major source of nitrogen (N) and organic matter for agricultural systems (Wisconsin 2024 Agricultural Statistics, 2024). When properly managed, manure can enhance soil fertility, improve soil structure, and support long‐term soil health by recycling valuable nutrients and maintaining or increasing soil organic carbon (C) (S. Liu et al., 2020). However, manure management can pose a threat to both water and air quality through nutrient losses via leaching, runoff, and gaseous emissions. Manure is a significant source of agricultural greenhouse gas (GHG) emissions—primarily methane (CH_4_) during storage and nitrous oxide (N_2_O) following field application (Habtewold et al., 2018; Jayasundara et al., 2016; Laubach et al., 2015). It is also an important source of nitric oxide (NO), a reactive nitrogen oxide that is formed as a by‐product of nitrification and as an intermediate in denitrification after manure application to soil (Burger & Horwath, 2013; Galbally & Roy, 1978; Russow et al., 2009; Veldkamp & Keller, 1997). Nitric oxide contributes to complex chemical reactions that drive smog formation, tropospheric ozone production, and acid rain (Warneck, 1999; Zhao et al., 2025). In parallel, ammonia (NH_3_) volatilization represents another major N loss pathway during both manure storage and land application (Bussink & Oenema, 1998; Huijsmans et al., 2001; Kavanagh et al., 2019). Ammonia volatilization reduces the fertilizer value of manure by depleting plant‐available N, leading to agronomic and economic inefficiencies. Moreover, volatilized NH_3_ contributes to atmospheric N deposition and the formation of fine particulate matter through secondary aerosol production, which poses risks to human health by exacerbating respiratory and cardiovascular conditions (Ti et al., 2019). Collectively, these challenges underscore the urgent need for manure management strategies that reduce emissions while preserving manure's agronomic value.

In response to these challenges, a range of innovations has emerged to tackle the twin goals of nutrient retention and emission reduction. International policy efforts, such as the UNECE's 2015 Framework Code on NH_3_ abatement (2015) and the UK's DEFRA Code of Good Agricultural Practice (2018), promote best practices like low‐emission housing, slurry acidification, and solid–liquid separation to curb NH_3_ and GHG outputs. Although several European countries have implemented policies and subsidies to encourage adoption of these technologies, similar regulatory support does not yet exist in the United States, where manure‐related GHG and NH_3_ emissions remain largely unregulated. Among the available methods, such as CH_4_ capture, improved application timing, and equipment upgrades, acidification and nitrification inhibitors (NIs) have emerged as particularly promising. Slurry acidification can significantly reduce NH_3_ and CH_4_ emissions during manure storage, while NIs serve to inhibit nitrification and mitigate gaseous N losses following field application (Kavanagh et al., 2019; Sokolov et al., 2019; Venterea et al., 2021).

Acidification lowers manure pH, shifting the chemical equilibrium from volatile NH_3_ gas toward the non‐volatile ammonium ion (NH_4_^+^), thereby suppressing NH_3_ volatilization and N loss (Fangueiro et al., 2015). Reducing pH below 6.5 greatly decreases the proportion of NH_3_ in solution, minimizing N loss during storage and after application (Fangueiro et al., 2015). Acidification can also reduce CH_4_ production during storage by inhibiting pH‐sensitive methanogens (Oertel et al., 2016; Sokolov et al., 2019). Effects on N_2_O emissions are more variable, likely because acidification alters the activity and composition of nitrifying and denitrifying microbes and the availability of substrates such as nitrate (NO_3_^−^) and dissolved organic carbon (DOC) (Cookson et al., 2007; Šimek & Cooper, 2002; Yao et al., 2011). Low pH reduces the activity of nitrite‐oxidizing bacteria (NOB) and N_2_O reductase, potentially decreasing NO_3_^−^ for denitrification but also limiting complete denitrification (Bakken et al., 2012; Jiménez et al., 2011; B. Liu et al., 2010; Šimek & Cooper, 2002). These mechanisms align with reports that acidified pig slurry suppressed early nitrification and lowered N_2_O emissions in sandy soils (Fangueiro et al., 2015), whereas acidified cattle slurry increased emissions due to higher soil NO_3_^−^ from nitric acid use (Velthof & Oenema, 1993). In other cases, such as Malique et al. (2021), acidification did not alter N_2_O emissions despite shifts in microbial function, including increased nitrite reductase gene transcripts.

NIs such as dicyandiamide (DCD) function by temporarily suppressing the first step of the nitrification process: the microbial oxidation of NH_4_^+^ to hydroxylamine (NH_2_OH) (Elrys et al., 2020; Ning et al., 2018). In agricultural soils, this reaction is primarily carried out by ammonia‐oxidizing bacteria (AOB), which use the enzyme ammonia monooxygenase (AMO) to initiate the conversion (Elrys et al., 2020; Shen et al., 2013). By inhibiting AMO activity, DCD slows the transformation of NH_4_^+^ to NH_2_OH, effectively limiting the subsequent production of nitrite (NO_2_^−^) and NO_3_^−^ and reducing the pool of N available for downstream processes like nitrifier denitrification and heterotrophic denitrification (Firestone & Davidson, 1989; Hollocher et al., 1981; Zhu et al., 2013). As a result, emissions of N_2_O and NO may be suppressed (Cameron et al., 2014; Venterea et al., 2021).

As acidification and NIs operate through different mechanisms to reduce N losses and GHG emissions, their efficacy is strongly shaped by soil properties and microbial context. Soils vary widely in their ability to buffer pH changes due to differences in organic matter content, mineralogy, and cation exchange capacity (CEC), which influence how long pH reductions persist after acidified manure application (Curtin & Rostad, 1997; Jarecki et al., 2008). Generally, soils with lower buffering capacities are more likely to experience sustained reductions in pH after acidified manure application, compared to soils with higher buffering capacities. These soil pH changes can significantly alter microbial activity, particularly among nitrifiers and denitrifiers, and shift the pathways and magnitudes of N transformations and associated N_2_O, NO, and carbon dioxide (CO_2_) emissions (Burth & Ottow, 1983; Oertel et al., 2016; Šimek & Cooper, 2002). Moreover, once manure is incorporated into soil, spatial heterogeneity in oxygen (O_2_), C, and N across soil microsites supports a mix of aerobic and anaerobic processes that influence GHG emission dynamics (Elrys et al., 2020; Y. Li et al., 2021; Wu et al., 2017). The performance of NIs such as DCD is also affected by soil texture, organic matter, and microbial interactions, generally decreasing in soils with higher CEC due to increased sorption of the inhibitor to mineral surfaces (Elrys et al., 2020; McGeough et al., 2016; Venterea et al., 2021). However, most existing research on DCD has focused on synthetic fertilizer systems, leaving key knowledge gaps regarding its behavior in manure‐amended, recently glaciated north‐temperate soils such as those in Wisconsin (Elrys et al., 2020; McGeough et al., 2016; Venterea et al., 2021). Together, these interrelated factors highlight the need for soil‐specific evaluations of manure management strategies to accurately predict their effectiveness in mitigating GHG emissions and gaseous N losses.

To address these gaps, we conducted a laboratory incubation experiment to evaluate how amending agricultural soils with acidified or DCD‐treated manure affects biogeochemical processes related to N and C cycling. We measured changes in GHG (N_2_O, CH_4_), CO_2_, and NO emissions and soil NH_4_^+^ and NO_3_^−^ concentrations across multiple soil types varying in texture and buffering capacity in Wisconsin. We hypothesized that (i) acidified manure would alter soil N dynamics, leading to increased N_2_O and NO emissions due to disrupted nitrification and denitrification pathways, and reduced CO_2_ emissions due to suppressed heterotrophic respiration. In contrast, we hypothesized that (ii) DCD‐treated manure would suppress nitrification and reduce N_2_O and NO emissions, without impacting microbial respiration (i.e., CO_2_ fluxes). Finally, we expected (iii) CH_4_ emissions to remain low and not differ among treatments, as aerobic incubation conditions and the addition of manure amendments would limit methanogenesis. Overall, this study aims to provide mechanistic insight into how acidification and nitrification inhibition of manure influence soil gas emissions and internal N transformations, informing best practices for reducing agricultural emissions while maintaining soil function.

**Core Ideas**

- Treating manure with a nitrification inhibitor significantly reduced soil N_2_O and NO emissions.
- Acidified manure increased N_2_O emissions in soils with low soil buffering capacity.
- Effect of acidification and nitrification inhibition of manure depends on soil characteristics.

## 2 MATERIALS AND METHODS

### 2.1 Soil and manure preparation

Soils were collected in fall 2024 from four agricultural fields representing dominant soil types across contrasting agricultural regions of Wisconsin, following crop harvest but prior to fall manure application. Sampling locations, previous crops, and soil characteristics are summarized in Table 1. Composite samples (0‐ to 20‐cm depth) were taken from multiple auger borings over tens of meters, air‐dried and sieved to 2 mm, and stored at 4°C until use. Soils were preincubated at 40% water holding capacity (WHC) for 7 days to minimize the respiration pulse associated with rewetting (Kieft et al., 1987).

**TABLE 1:** Soil and manure slurry properties.

Properties	Plano 1	Plano 2	Plainfield	Ringwood	M	M + NI	M + A
Location	Leeds, WI	Leeds, WI	Hancock, WI	Arlington, WI			
Previous crop	Corn, grain	Pasture	Corn, grain	Corn, grain			
Soil texture	Silt loam	Silt loam	Sand	Silt loam			
% Clay	18	21	4	23			
% Silt	76	74	7	66			
% Sand	6	5	89	11			
WHC, g kg^−1^	616	535	217	493			
DM, g kg^−1^					100	100	100
pH	6.17	6.39	6.28	7.24	7.07	6.99	5.50
Buffering capacity, mmolc kg^−1^ pH^−1^	59.2	44.7	13.8	59.6			
TC, g kg^−1^	39.2	29.1	4.7	19.1	23.5	30.4	33.7
DOC, mg kg^−1^	224.3	152.4	79.4	116.2			
TN, g kg^−1^	3.7	2.7	0.5	1.8	0.8	1.3	1.2
NH_4_^+^‐N, mg kg^−1^	3.2	1.1	0.3	0.8	548	578	544
NO_3_^—^N, mg kg^−1^	67.1	30.9	18.0	23.9	1.1	1.0	0.5
CEC, cmol_c_ per 100 g soil	20.7	17.1	2.9	13.8			

*Note*: Manure data are expressed on a fresh weight basis.

Abbreviations: CEC, cation exchange capacity; DM, dry matter; DOC, dissolved organic carbon; M, manure; M + A, manure + acid; M + NI, manure + nitrification inhibitor; NH4+‐N, ammonium‐nitrogen; NO3^−^‐N, nitrate‐nitrogen; TC, total carbon; TN, total nitrogen; WHC, water holding capacity.

Dairy manure was collected from the UW–Madison Arlington Dairy Research Center and stored at 4°C. Dry matter content was determined by oven‐drying at 105°C to constant weight. Seven days before the experiment, manure was homogenized and divided into three subsamples for treatment. Two were diluted with deionized (DI) water to 90% moisture to generate slurry. The third was acidified: after dilution to 88% moisture, 9.6% (v/v) sulfuric acid was added incrementally (1 mL additions) with stirring until pH 5.5 was reached. pH was rechecked after 10 min for stability, and DI water was added to adjust final moisture to 90%. All manure slurries were stored at 4°C until application. Inorganic N (NH_4_^+^ and NO_3_^−^) content was measured colorimetrically (Doane & Horwáth, 2003; Verdouw et al., 1978) immediately before use. Slurry pH was also rechecked at that time. Total carbon (TC) and total nitrogen (TN) were determined using an elemental analyzer (Elementar Analysensysteme GmbH) after freeze‐drying and ball‐milling. Manure characteristics are listed in Table 1.

### 2.2 Soil incubation experiment

Preincubated soils (200 g dry mass equivalent) were placed into glass jars (473 mL) and received one of four manure treatments: no‐manure, manure, manure + NI, or manure + acid. Manure‐amended treatments received slurry at a rate of 60 mg NH_4_^+^‐N kg^−1^ soil. For the manure + NI treatment, DCD was added to the manure at 15% of the manure NH_4_^+^‐N mass on the day of application. This approach was previously reported in similar incubation studies to approximate effective inhibitor doses (i.e., equivalent to ∼10–30 kg DCD ha^−1^, a range shown to reduce nitrification in field trials) (Cahalan, Ernfors, et al., 2015; Cahalan, Minet, et al., 2015; Elrys et al., 2020). To simulate field injection and surface overflow, manure was applied in two layers: half the soil was placed in the jar and manured, followed by the remaining soil and a second manure application. DI water was added to adjust soil moisture to 60% of WHC. For the no‐manure treatments, the same procedure was followed, but only DI water was applied in place of manure. The experiment was arranged in a completely randomized block design and incubated at 22°C for 28 days. Each treatment was replicated three times for gas measurements and for the final soil sampling. Seven additional sampling events (also triplicated) were included for destructive soil NH_4_^+^/NO_3_^−^ sampling, totaling 384 jars. Soil moisture was maintained at target levels by weighing jars every other day and replacing water lost to evaporation.

### 2.3 Gas sampling and analysis

Gas fluxes were measured on days 1, 2, 3, 5, 7, 9, 14, 21, and 28 of the incubation period. Carbon dioxide (CO_2_) and CH_4_ fluxes were measured using a LI‐COR 7810 trace gas analyzer, while N_2_O fluxes were measured using a LI‐COR 7820 analyzer. Nitric oxide (NO) fluxes were quantified using a Model 405 nm NO_2_/NO/NO*_x_* Monitor (2B Technologies). We used a steady‐state flow‐through chamber approach (Hall et al., 2018), whereby ambient room air was flushed through each jar for at least 2 min at 3.0 L min^−1^ via a pump and mass flow controller. The three gas analyzers described above were plumbed in parallel downstream of the mass flow controller, with excess flow vented to the room. Incubation jars were fitted with custom stainless‐steel lids equipped with Viton gaskets, opaque Teflon tubing, and stainless‐steel Swagelok fittings to create airtight, low‐reactivity sampling systems.

### 2.4 Soil extraction and analysis

Soil inorganic N (NH_4_^+^‐N and NO_3_^−^‐N) contents were analyzed on days 0, 1, 3, 5, 7, 14, 21, and 28 after application of manure. At each sampling point, soils were extracted with 2 M KCl using a 4:1 extractant‐to‐soil mass ratio. Extractions were conducted by shaking the mixture for 1 h, followed by centrifugation at 16,100 g for 2 min. The supernatant was immediately transferred to cuvettes for analysis. NH_4_^+^ and NO_3_^−^ contents were analyzed using colorimetric methods (Doane & Horwáth, 2003; Verdouw et al., 1978).

For the initial soil samples, soil particle size distribution was determined using a modified pipette method, and soil texture was classified according to the USDA soil texture triangle (Miller & Miller, 1987; Soil Science Division Staff 2017). WHC was measured using the funnel and filter paper drainage method (Nelson et al., 2024). Soil pH was measured in a 1:2.5 soil‐to‐DI water suspension using an Orion Dual Star pH/ISE meter (Thermo Scientific). pH buffering capacity was determined via acid‐base titration following the method of Wang et al. (2015). TC and TN were analyzed with an elemental analyzer (Elementar Analyzensysteme GmbH) after samples were ground using a ball mill. DOC was quantified in 2 M KCl soil extracts (4:1 extractant‐to‐soil ratio, 1‐h shake) using a Shimadzu TOC‐L series analyzer (Shimadzu Corporation) (Jones & Willett, 2006). Extracts were filtered through 0.3 µm filters (Advantec GF‐75) prior to analysis. Cation exchange capacity (CEC) was determined using the ammonium acetate method (Munera‐Echeverri et al., 2018; Nel et al., 2023; Sumner & Miller, 1996).

### 2.5 Calculations

Gas fluxes were calculated via mass balance as the difference in trace gas mole density between ambient room air and incubation jar outlet (headspace emissions) over a 2‐min measurement period, multiplied by the inlet flow rate (3.0 L min^−1^). Outlet concentrations of measured gases typically stabilized between 30 and 50 seconds after the start of sampling; therefore, the average concentration data collected between 55 and 115 seconds were used to calculate gas fluxes. Cumulative gas emissions (CO_2_‐C, CH_4_‐C, N_2_O‐N, and NO‐N) were calculated using trapezoidal integration of daily gas fluxes, with the assumption that the gas flux measured at each time point was representative of the average daily flux.

Net N mineralization (NNmin) during specific time intervals was calculated using the following formula:

(1) $$\begin{array}{ccc} \mathrm{NNmin}\;\left(\mathrm{mg}\;N\;kg^{-1}\right) & = & \left[\mathrm{TI}N_{T}\left(t_{f}\right)-\mathrm{TI}N_{C,\mathrm{avg}}\left(t_{f}\right)\right]\\ & & -\;\left[\mathrm{TI}N_{T}\left(t_{i}\right)-\mathrm{TI}N_{C,\mathrm{avg}}\left(t_{i}\right)\right] \end{array}$$

where TIN_T_ and TIN_C, avg_ correspond to the total inorganic N (NH_4_^+^‐N and NO_3_^−^‐N) content in the manured treatment and the no‐manure control at the beginning (t_i_) and end (t_f_) of the corresponding period. TIN_C, avg_ represents the average of the three no‐manure replicates.

Net N mineralization rate (NNminR) was further calculated as follows:

(2) $$\mathrm{NNminR}\;\left(\mathrm{mg}\;N\;kg^{-1}\mathrm{da}y^{-1}\right)=\frac{\mathrm{NNmin}}{\mathrm{Period}}$$

where Period is the time in days of the NNmin period.

Cumulative mineralized N (CminN) was calculated as follows:

(3) $$\mathrm{CminN}\;\left(\mathrm{mg}\;N\;kg^{-1}\right)=\left[\mathrm{TI}N_{T}\left(t_{28}\right)\right]-\left[\mathrm{TI}N_{T}\left(t_{0}\right)\right]$$

where TIN_T_ corresponds to the total inorganic N (NH_4_^+^‐N and NO_3_^−^‐N) content in the manured treatment at the beginning (*t*_0_) and end (*t*_28_) of the incubation.

Net nitrification (NNit) during specific time intervals was calculated using the following formula:

(4) $$\begin{array}{ccc} \mathrm{NNit}\;\left(\mathrm{mg}\;N\;kg^{-1}\right) & = & \left[N_{T}\left(t_{f}\right)-N_{C,\mathrm{avg}}\left(t_{f}\right)\right]\\ & & -\;\left[N_{T}\left(t_{i}\right)-N_{C,\mathrm{avg}}\left(t_{i}\right)\right] \end{array}$$

where N_T_ and N_C, avg_ correspond to the NO_3_^−^‐N content in the manured treatment and the no‐manure control at the beginning (t_i_) and end (t_f_) of the corresponding period. N_C, avg_ represents the average of the three no‐manure replicates.

Net nitrification rate (NNitR) was calculated as:

(5) $$\mathrm{NNitR}\;\left(\mathrm{mg}\;N\;kg^{-1}\mathrm{da}y^{-1}\right)=\frac{\mathrm{NNit}}{\mathrm{Period}}$$

where Period is the time in days of the NNit period.

Cumulative nitrified NO_3_^−^ (CnitN) was calculated as:

(6) $$\mathrm{CnitN}\;\left(\mathrm{mg}\;N\;kg^{-1}\right)=\left[N_{T}\left(t_{28}\right)\right]-\left[N_{T}\left(t_{0}\right)\right]$$

where N_T_ corresponds to the total NO_3_^−^‐N content in the manured treatment at the beginning (*t*_0_) and end (*t*_28_) of the incubation.

### 2.6 Statistical analysis

All analyses were conducted in R (v4.4.2, R Core Team, 2024). Data processing and visualization used the tidyverse, ggplot2, and ggtext packages (Wickham, 2016; Wickham et al., 2019; Wilke & Wiernik, 2022). Outliers in CH_4_ flux measurements (*n* = 5) were identified using the 1.5× interquartile range rule and removed prior to analysis. Net N mineralization and nitrification rates were analyzed with a three‐way linear model including treatment, soil type, day range, and their interactions as fixed effects. Cumulative mineralized N, nitrified NO_3_^−^, and gas emissions were analyzed using two‐way models with treatment, soil type, and their interaction. Model assumptions were assessed using residual plots (plot.lm) and the DHARMa package (Hartig, 2024). To meet assumptions of normality and homoscedasticity, N_2_O data were log‐transformed and NO data were square‐root transformed. Type III analyses of variance (ANOVAs) were performed using the car package (Fox & Weisberg, 2019), and post hoc comparisons were made with estimated marginal means (emmeans) (Lenth, 2025), using compact letter displays to denote significant differences (*α* = 0.05). Corresponding *p*‐values from pairwise comparisons were also obtained from the emmeans results and reported where relevant. Coefficient estimates, standard errors, and *p*‐values for fixed effects were obtained directly from fitted linear models using the summary() function in R. Reported coefficients represent model estimates on the transformed response scale (log‐transformed for N_2_O, square root‐transformed for NO, and untransformed for all other response variables).

## 3 RESULTS

### 3.1 Dynamics of soil inorganic N concentrations

**FIGURE 1:**
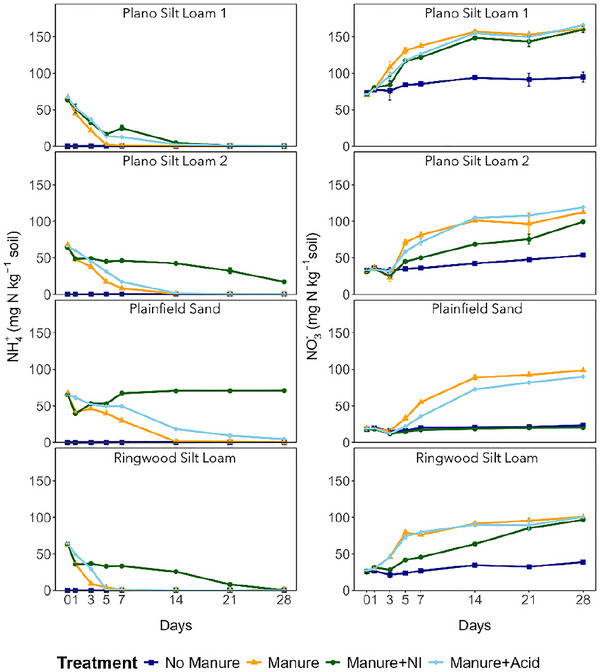
Dynamics of soil NH_4_^+^ and NO_3_^−^ concentrations during the incubation across different soils amended with various manure treatments. Error bars indicate standard error (*n* = 3).

Soil NH_4_^+^ concentrations declined rapidly within the first week in soils amended with manure or manure + acid, though the decline was slightly delayed in the latter (Figure 1). In contrast, the manure + NI treatment enhanced NH_4_^+^ retention, especially in Plano Silt Loam 2 and Plainfield Sand, which maintained concentrations above 10 mg N kg^−1^ soil through day 28. The concentrations of NO_3_^−^ increased in all manure‐treated soils except for Plainfield Sand under manure + NI, where little accumulation was observed. The NI treatment generally delayed NO_3_^−^ accumulation in Plano Silt Loam 2 and Ringwood Silt Loam, while the concentrations of NO_3_^−^ slightly increased in no‐manure controls.

### 3.2 Net N mineralization and nitrification

**FIGURE 2:**
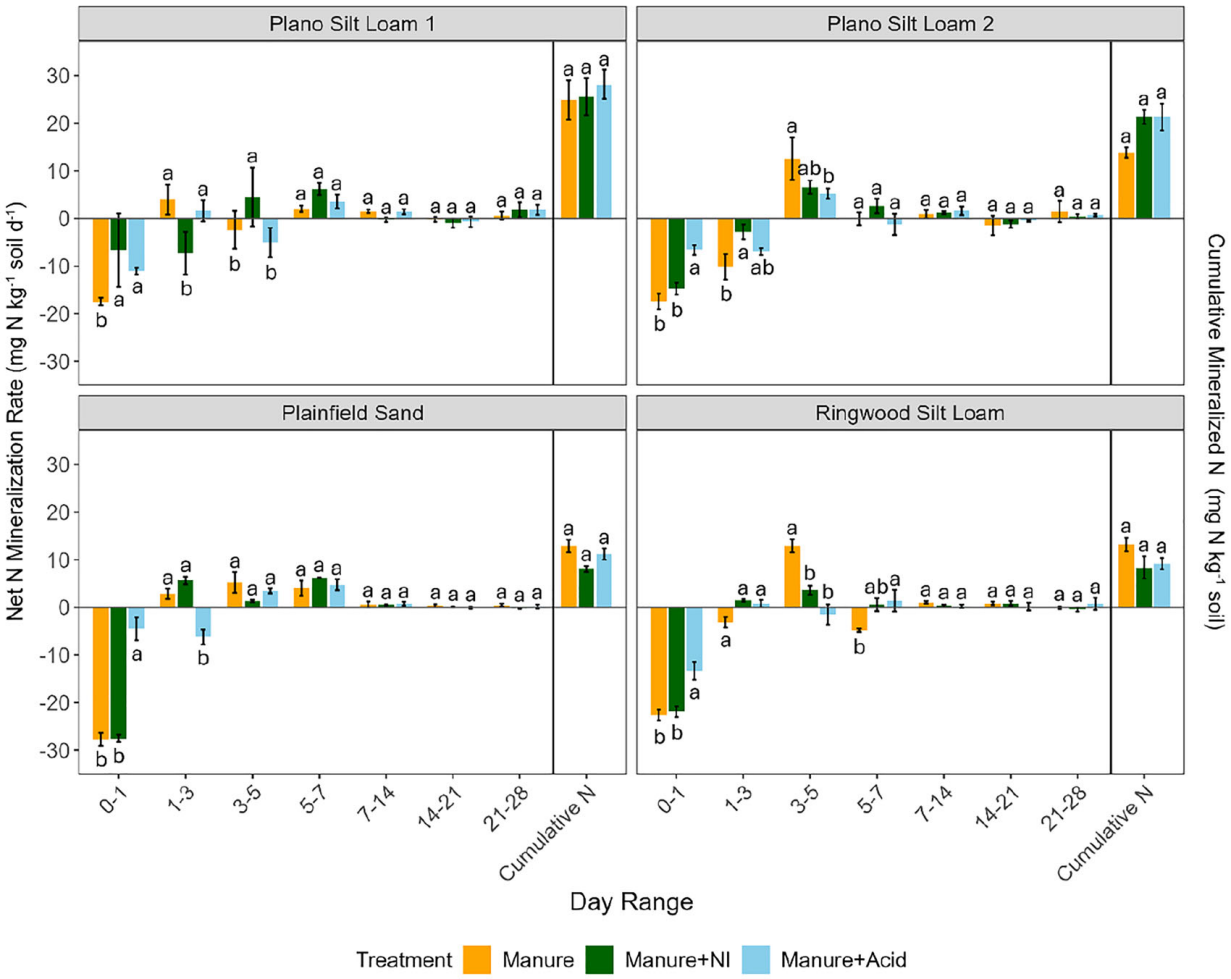
Net N mineralization rates for each incubation period and cumulative mineralized N over the entire incubation course. Lowercase letters indicate significant differences among manure treatments within each soil type and incubation period. Significance was determined at *α* = 0.05 (*n* = 3).

Net N mineralization and nitrification rates varied significantly by soil type, manure treatment, incubation period, and their interactions (Table 2; Table S2). During the incubation, the manure and manure + NI treatments showed the lowest net N mineralization rates on Days 0–1 across most soils, followed by a gradual increase over time (Figure 2; Table S3). For the manure + acid treatment, the timing of minimum net N mineralization rates varied by soil: Days 1–3 in Plano Silt Loam 2 and Plainfield Sand; Days 0–1 in the others. Despite these temporal differences, cumulative mineralized N did not differ significantly (*p *> 0.05) among manure treatments; however, soil type had a stronger influence than manure treatment, with Plano Silt Loam 1 consistently showing the highest values among all the soils (Figure 2; Tables S1 and S3).

**TABLE 2:** Summary of ANOVA results examining the effects of soil type, manure treatment, incubation period, and their interactions on net N mineralization rate, cumulative mineralized N, net nitrification rate, and cumulative nitrified NO_3_^−^.

Factor/interaction	Net N mineralization rate	Cumulative mineralized N	Net nitrification rate	Cumulative nitrified NO_3_^−^
Soil type	<0.001	<0.001	0.011	0.001
Manure treatment	<0.001	0.586	0.724	<0.001
Manure treatment × soil type	<0.001	0.094	0.758	<0.001
Incubation period	<0.001	–	<0.001	–
Manure treatment × incubation period	<0.001	–	<0.001	–
Incubation period × soil type	<0.001	–	<0.001	–
Incubation period × soil type × manure treatment	<0.001	–	<0.001	–

*Note*: Reported values are *p*‐values. Hyphens mark factors or interactions that are not applicable.

**FIGURE 3:**
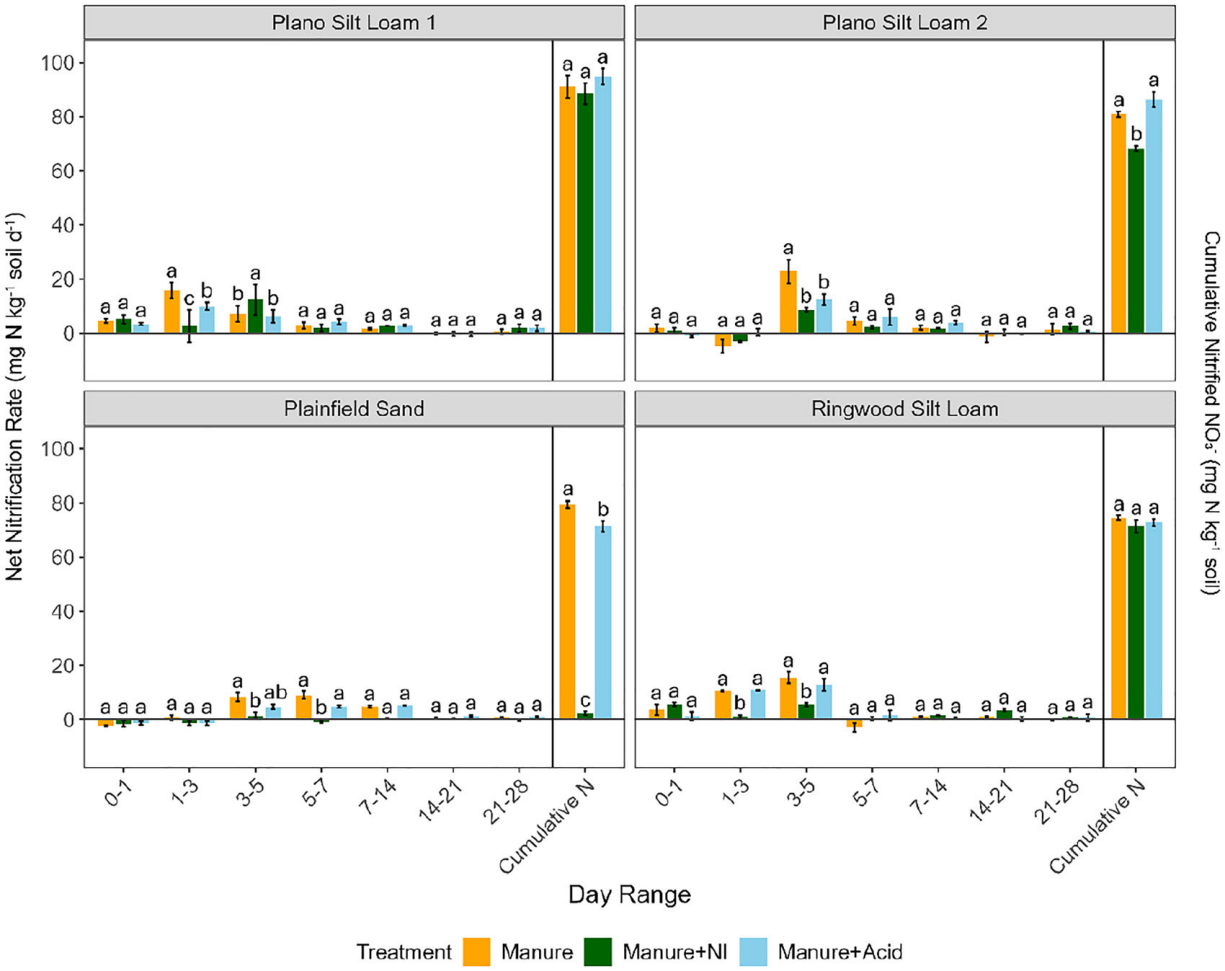
Net nitrification rates for each incubation period and cumulative nitrified NO_3_^−^ over the entire incubation course. Lowercase letters indicate significant differences among manure treatments within each soil type and incubation period. Significance was determined at *α* = 0.05 (*n* = 3).

Nitrification was greatest under the untreated manure, peaking during Days 1–7 (Figure 3; Table S4). The manure + NI treatment delayed peak nitrification to Days 3–5 in most soils, except for Ringwood Silt Loam, which peaked on Days 0–1. The manure + acid treatment followed similar trends but peaked later in Plainfield Sand (Days 7–14).

### 3.3 Gas emissions

**FIGURE 4:**
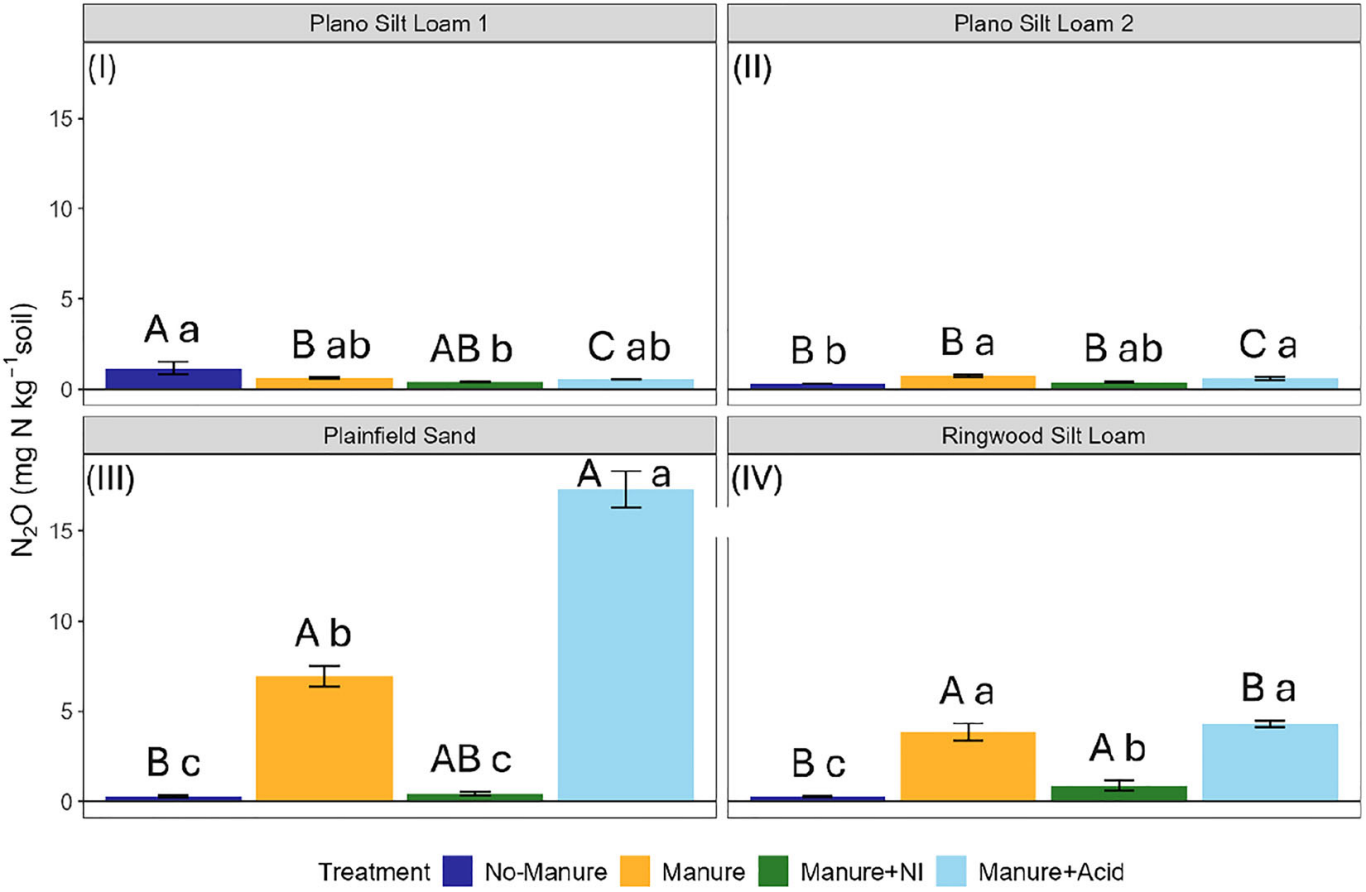
Cumulative N_2_O emissions from four soils following a 28‐day incubation with different manure amendments. Each panel represents a different soil type: (I) Plano Silt Loam 1, (II) Plano Silt Loam 2, (III) Plainfield Sand, and (IV) Ringwood Silt Loam. Within each panel, bars indicate mean cumulative N_2_O emissions for the four amendments: no‐manure, manure, manure + NI (nitrification inhibitor), and manure + acid (acidified manure). Error bars represent standard error (*n* = 3). Uppercase letters above bars denote differences among soils within each treatment only; lowercase significance letters above bars denote differences among treatments within each soil type only, based on estimated marginal means (*α* = 0.05) with Tukey‐adjusted pairwise comparisons.

Cumulative N_2_O emissions were significantly affected by soil type, manure treatment, and their interaction (Table 3; Table S5). In general, the manure and manure + acid treatments led to the highest emissions, while manure + NI suppressed them (Figure 4). In Plainfield Sand, manure + acid produced the highest emissions, exceeding all other treatments. In Ringwood Silt Loam, both manure and manure + acid produced higher emissions than no‐manure or manure + NI treatments. In Plano Silt Loam 2, the untreated manure (*p *= 0.006) and manure + acid (*p *= 0.045) treatments significantly increased emissions compared to the no‐manure control. In Plano Silt Loam 1, the no‐manure control had higher emissions than manure + NI, with other treatments being intermediate.

**TABLE 3:** Summary of ANOVA results examining the effects of soil type, manure amendment, and their interaction on cumulative gas emissions (N_2_O, NO, CH_4_, and CO_2_).

Factor/interaction	N_2_O cumulative emissions	NO cumulative emissions	CH_4_ cumulative emissions	CO_2_ cumulative emissions
Soil type	<0.001	0.747	<0.001	0.044
Manure amendment	0.007	0.003	0.173	0.019
Manure amendment × soil type	<0.001	0.495	<0.001	0.028

*Note*: Reported values are *p*‐values.

**FIGURE 5:**
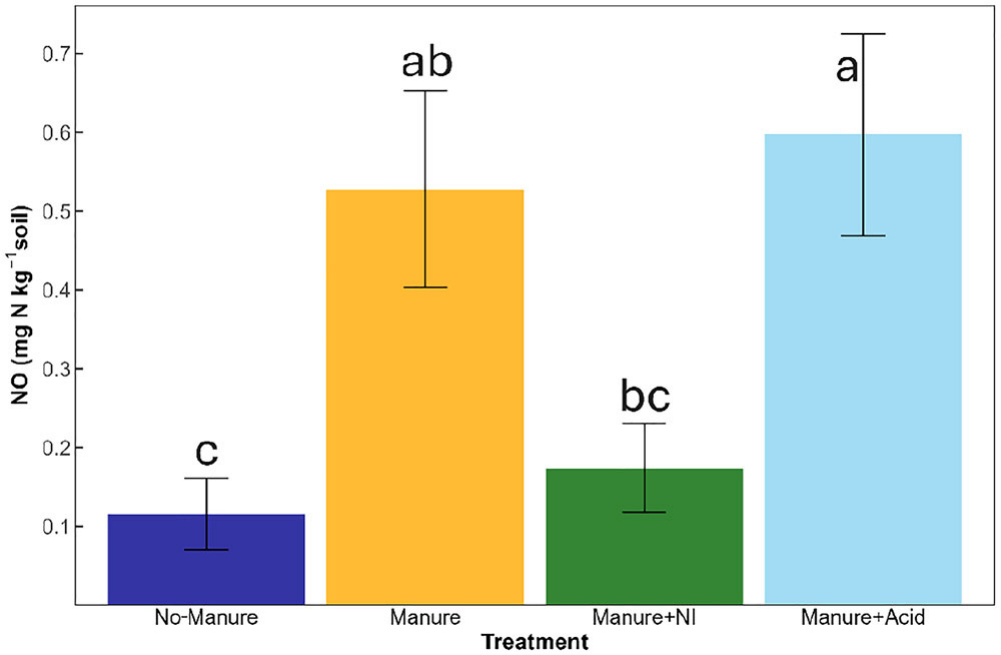
Cumulative NO emissions across all soils following a 28‐day incubation with different manure amendments. Each bar represents the mean cumulative NO emissions across soil types within each manure treatment: No‐Manure, Manure, Manure + NI (nitrification inhibitor), and Manure + Acid (acidified manure). Error bars represent standard error (*n* = 12). Significance letters above bars denote differences among treatments based on estimated marginal means (*α* = 0.05) with Tukey‐adjusted pairwise comparisons.

Cumulative NO emissions differed significantly among manure treatments but not soil types (Table 3; Table S5). Manure + acid produced the highest emissions, significantly greater than no‐manure (*p *< 0.001) and manure + NI (*p *= 0.009) (Figure 5). Emissions under manure alone were intermediate.

**FIGURE 6:**
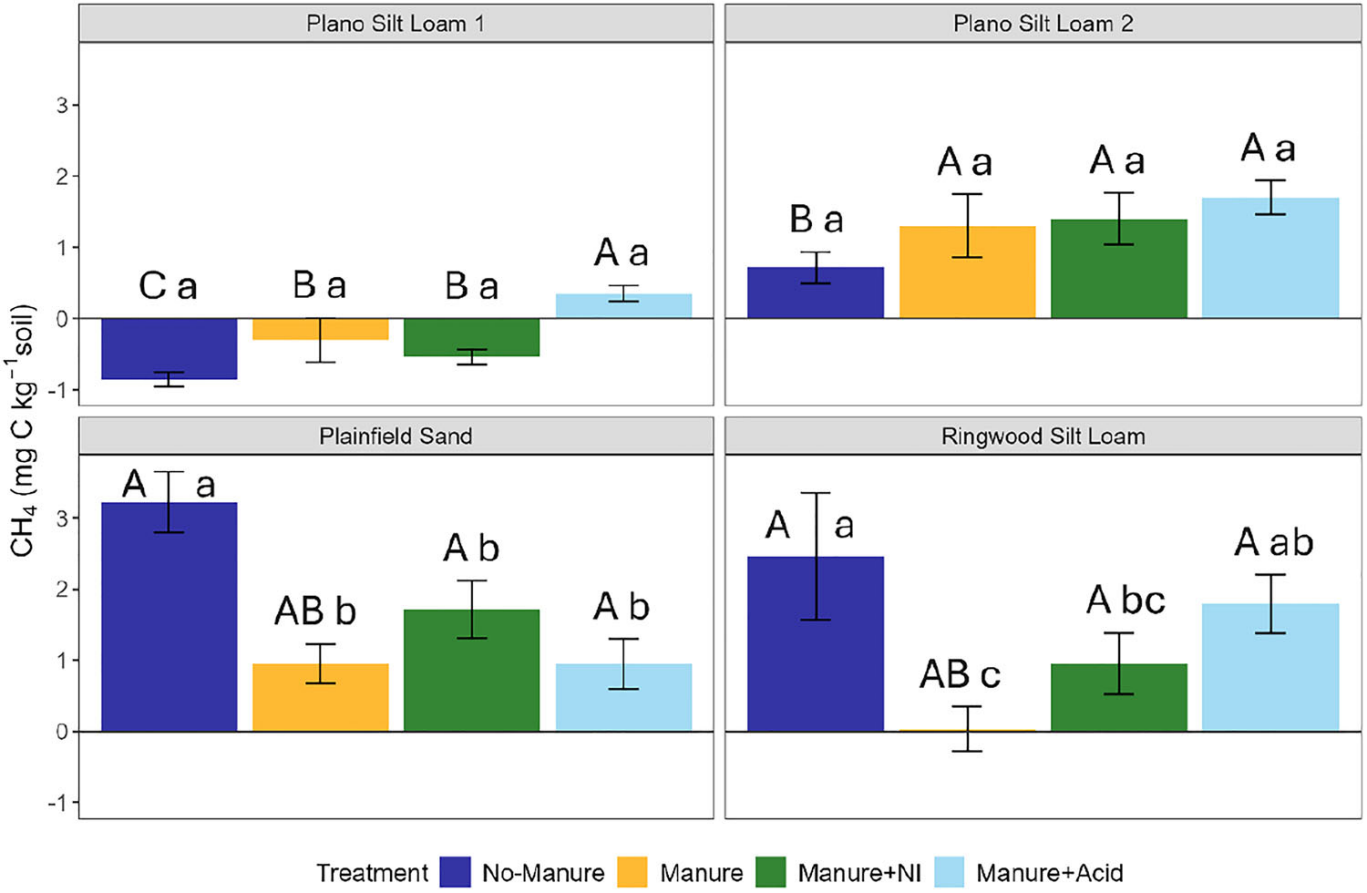
Cumulative CH_4_ emissions from four soils following a 28‐day incubation with different manure amendments. Each panel represents a different soil type: (I) Plano Silt Loam 1, (II) Plano Silt Loam 2, (III) Plainfield Sand, and (IV) Ringwood Silt Loam. Within each panel, bars indicate mean cumulative CH_4_ emissions for the four manure treatments: No‐Manure, Manure, Manure + NI (nitrification inhibitor), and Manure + Acid (acidified manure). Error bars represent standard error (*n* = 3). Uppercase letters above bars denote differences among soils within each treatment only; lowercase significance letters above bars denote differences among treatments within each soil type only, based on estimated marginal means (*α* = 0.05) with Tukey‐adjusted pairwise comparisons.

The emissions of CH_4_ varied by soil type, with some treatment effects (Table 3; Table S5). No significant differences between treatments were observed in the two Plano Silt Loam soils (*p *> 0.05), although net CH_4_ consumption (negative emissions) occurred in some cases (i.e., manure + NI in Plano Silt Loam 1). The no‐manure treatment in Plano Silt Loam 1 exhibited significantly lower (*p* < 0.05) CH_4_ emissions than the corresponding treatments in other soils (Figure 6). In Plainfield Sand and Ringwood Silt Loam, CH_4_ emissions were significantly higher (*p *< 0.05) under the no‐manure treatment than with manure or manure + NI.

**FIGURE 7:**
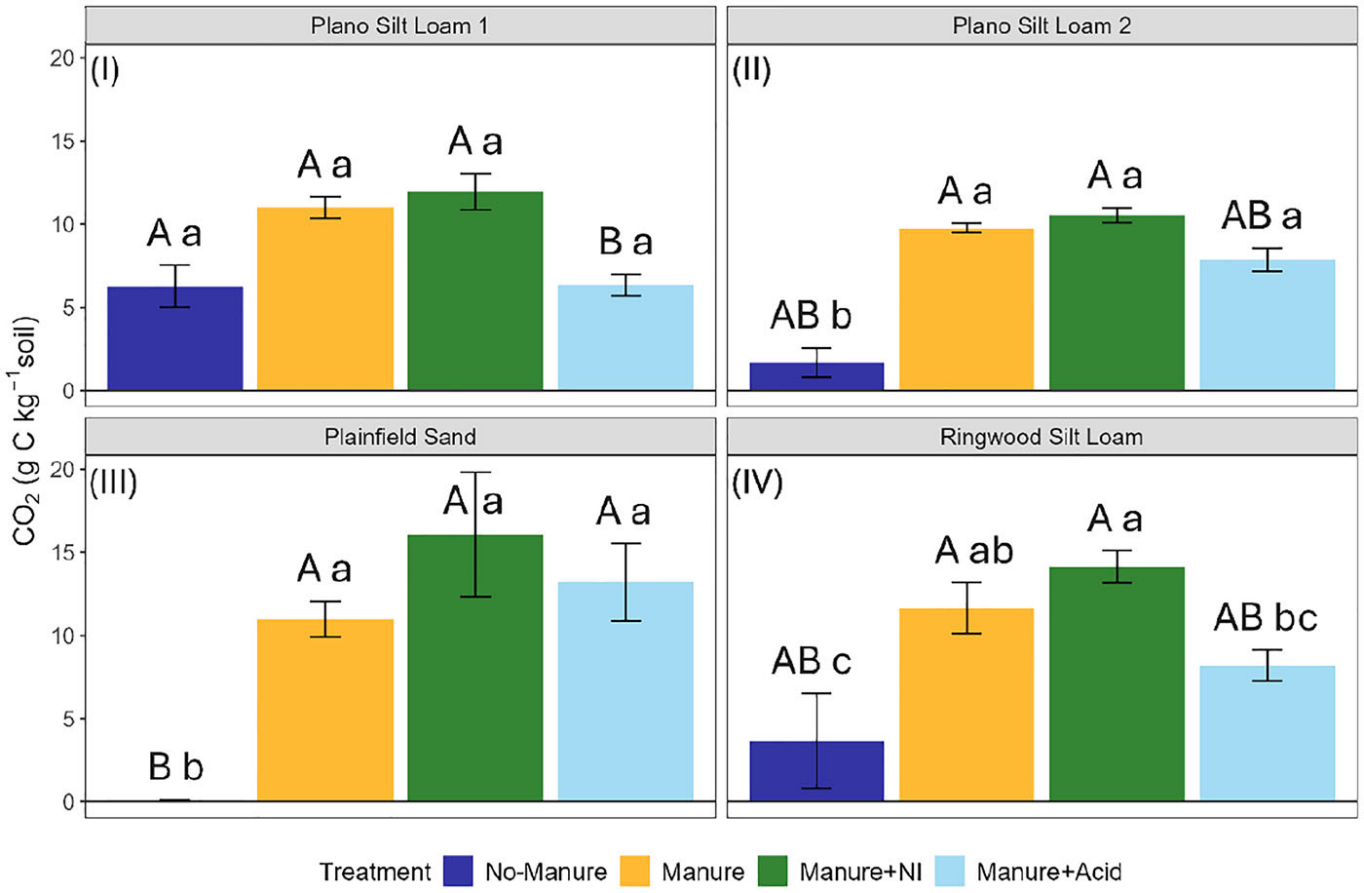
Cumulative CO_2_ emissions from four soils following a 28‐day incubation with different manure amendments. Each panel represents a different soil type: (I) Plano Silt Loam 1, (II) Plano Silt Loam 2, (III) Plainfield Sand, and (IV) Ringwood Silt Loam. Within each panel, bars indicate mean cumulative CO_2_ emissions for the four amendments: No‐Manure, Manure, Manure + NI (nitrification inhibitor), and Manure + Acid (acidified manure). Error bars represent standard error (*n* = 3). Uppercase letters above bars denote differences among soils within each treatment only; lowercase significance letters above bars denote differences among treatments within each soil type only, based on estimated marginal means (*α* = 0.05) with Tukey‐adjusted pairwise comparisons.

Cumulative CO_2_ emissions were significantly affected by soil type, manure treatment, and their interaction (Table 3; Table S5). Manure‐amended soils generally had greater CO_2_ emissions than the no‐manure control (Figure 7). The manure treatments did not differ significantly (*p *> 0.05) in CO_2_ emissions in any soil except the Ringwood Silt Loam, where the manure + acid treatment had significantly (*p *= 0.048) lower emissions than the manure + NI treatment.

## 4 DISCUSSION

### 4.1 Soil N dynamics and gaseous N emissions

Manure amendments influenced soil N cycling and gaseous N losses largely through early differences in NH_4_^+^ availability and nitrification activity, although they had minimal impact on cumulative mineralized N over the 28‐day incubation. Acidification of manure (manure + acid treatment) enhanced NH_4_^+^ retention in soils during the initial stage of the experiment (Figure 2; Table S3). This effect is consistent with the known chemical mechanism by which acidified manure lowers soil pH, thereby shifting the NH_4_^+^/NH_3_ equilibrium toward the non‐volatile NH_4_^+^ form and reducing NH_3_ volatilization losses (Ellersiek & Olfs, 2024; Fangueiro et al., 2017; Kai et al., 2008). Our results support this mechanism, as all the studied soils had significantly greater (*p* < 0.05) NH_4_^+^ retention between days 0 and 1 in the manure + acid treatment compared to the untreated manure (Figure 2; Table S3). These results suggest that even a transient reduction in pH following manure application can meaningfully alter early N dynamics by limiting NH_3_ volatilization and preserving plant‐available NH_4_^+^.

Despite the early differences in NH_4_^+^ retention among treatments, there were no significant differences (*p *> 0.05) in cumulative mineralized N over the full 28‐day incubation (Figure 2; Table S3). This suggests that the short‐term pH decreases from acidification were unlikely to have substantially influenced the overall activity of heterotrophic microbes responsible for N mineralization—microbial groups generally less sensitive to pH fluctuations than ammonia‐ and nitrite‐oxidizing populations (Curtin et al., 1998). In contrast, NI addition may have helped maintain a more stable soil pH by suppressing acidifying nitrification; however, this stabilization did not correspond to measurable changes in heterotrophic microbial activity, as inferred from cumulative CO_2_ emissions (Figures 3 and 7; Table S4). Moreover, the organic matter supplied by the manure likely provided ample DOC and dissolved organic nitrogen as substrate to sustain microbial activity across all treatments, buffering any temporary inhibitory effects of lower pH (Curtin et al., 1998). While cumulative mineralized N was unaffected, treatment‐induced differences in early N turnover, particularly in nitrification, resulted in distinct temporal patterns of inorganic N pools (Figure 3; Table S4). These shifts in substrate availability likely contributed to the observed variation in N gas fluxes over time.

Across all soils, early nitrification activity was strongly influenced by treatment. The use of NI consistently suppressed early nitrification rates (Figure 3; Table S4), resulting in significantly (*p *< 0.001) lower cumulative N_2_O emissions compared to untreated manure and acidified manure in the Plainfield Sand and Ringwood Silt Loam (Figure 4). These findings align with prior studies showing that NIs such as DCD reduce N_2_O emissions by slowing the microbial oxidation of NH_4_^+^ (Qiao et al., 2015; Wu et al., 2017; Zhu‐Barker et al., 2015). DCD functions by binding to the AMO enzyme used by AOB, thereby directly inhibiting NH_4_^+^ oxidation and subsequently reducing NO_3_^−^ accumulation and nitrifier O_2_ consumption (Elrys et al., 2020; Ning et al., 2018). This in turn limits the conditions for N_2_O production via nitrifier denitrification and heterotrophic denitrification, microbiological processes triggered under low O_2_ and high NO_3_^−^ conditions (Zhu et al., 2013). In the first days of the incubation, large CO_2_ fluxes suggest rapid O_2_ consumption by heterotrophic microbes, potentially forming anaerobic microsites within the soil. During this same period, DCD also slowed the accumulation of soil NO_3_^−^, potentially within these microsites before conditions became favorable for heterotrophic denitrification, thereby reducing cumulative N_2_O emissions (Zhu et al., 2013). In contrast, rapid NH_4_^+^ oxidation in the untreated manure treatment likely increased NO_3_^−^ accumulation before these microsites became anaerobic, leading to greater cumulative N_2_O emissions compared to the manure treated with NI (Elrys et al., 2020; Ning et al., 2018).

Given that incubation temperature was uniform across treatments, variability in DCD performance among soils may be partly explained by differences in soil physical and chemical properties. Prior research indicates that the efficacy of NIs tends to decline in soils with higher clay content and organic matter due to increased sorption of the inhibitor to mineral surfaces (McGeough et al., 2016; Venterea et al., 2021). In this study, the efficacy of NI (indicated by a decrease in cumulative nitrified N) was highest in the Plainfield Sand, followed by the Plano Silt Loam 2 and Ringwood Silt Loam, and was lowest in the Plano Silt Loam 1 (Figure 3; Table S4). This is likely due to the Plainfield Sand having lower soil organic C and coarser textures, contributing to decreased DCD sorption. The Plano Silt Loam 2 and Ringwood Silt Loam had intermediate soil organic C and fine mineral particles, while the Plano Silt Loam 1 had the highest soil organic C (Table 1). Notably, in the Plainfield Sand, cumulative nitrified N in the manure + NI treatment was minimal (2.28 mg N kg^−1^), indicating strong nitrification suppression with DCD in this soil, which had low soil organic C and clay content (Figure 3; Table S4).

Manure acidification also influenced N gas emissions, though the patterns were more variable across soil types. In the Plainfield Sand, manure acidification significantly (*p *= 0.003) increased N_2_O emissions compared to untreated manure, while no such difference was observed in the Ringwood Silt Loam (*p* = 0.955) (Figure 4). This contrast likely reflects the differences in the soils’ capacities to buffer acidification. The Plainfield Sand, with a lower baseline pH (6.28) and limited buffering capacity (Table 1), may have experienced a greater and more persistent pH reduction, sufficient to inhibit N_2_O reductase (nosZ) activity, the enzyme responsible for reducing N_2_O to N_2_ during denitrification (Šimek & Cooper, 2002). In contrast, the Ringwood Silt Loam (pH 7.2) with a much higher buffering capacity (Table 1) may have experienced less pH change when acidified manure was introduced, preserving similar nosZ activity compared to the untreated manure. These findings are consistent with prior work showing that nosZ activity declines under low pH, leading to increased N_2_O accumulation (Bakken et al., 2012; B. Liu et al., 2010; Šimek & Cooper, 2002).

Cumulative NO emissions also differed among treatments in ways consistent with shifts in nitrification and pH‐mediated microbial processes. Among treatments, soils that received acidified manure produced significantly (*p* < 0.01) higher cumulative NO emissions than the no‐manure and manure + NI treatments, while the NO emissions from the soils that received untreated manure were not significantly different (*p* > 0.05) from the other manure treatments (Figure 5). This pattern suggests that the application of acidified manure may have promoted conditions conducive to NO_2_^−^ accumulation, a process known to favor both biotic and abiotic NO production, likely through the inhibition of NOB under low pH (Jiménez et al., 2011; Russow et al., 2009; Zhu‐Barker et al., 2015). While significant (*p* < 0.05) reductions in nitrification rates by acidified manure were only observed during two soil‐ and time‐specific windows (Days 1–3 in Plano Silt Loam 1 and Days 3–5 in Plano Silt Loam 2) (Figure 3; Table S4), it is important to note that nitrification rate was determined as the sum of NO_2_^−^ and NO_3_^−^ produced in a given time. As such, inhibited NO_2_^−^ oxidation under low pH could have allowed NO_2_^−^ to accumulate without reducing overall nitrification values.

In contrast, the low NO emissions from the soils that received manure + NI (Figure 5) may be attributed not only to reduced NO_2_^−^ production but also to the continued activity of NOB. By selectively inhibiting AMO in AOB, DCD slows the formation of NO_2_^−^ from NH_4_^+^ oxidation (Elrys et al., 2020; Ning et al., 2018). NOB, which can efficiently oxidize NO_2_^−^ even at low substrate concentrations, remain active; thus, any residual NO_2_^−^ that is produced can be rapidly oxidized to NO_3_^−^, preventing its accumulation (Nowka et al., 2015; Okabe et al., 1999; Schramm et al., 1998). With NO_2_^−^ concentrations kept low, there is less substrate available for both biotic and abiotic conversion of NO_2_^−^ to NO, which may partly explain the lower cumulative NO emissions observed in the soils that received manure with NI (Ning et al., 2018; Venterea et al., 2021). Furthermore, AMO inhibition reduces O_2_ consumption in microsites, potentially preserving O_2_ for NOB activity (Laanbroek & Gerards, 1993; Venterea et al., 2021). This effect could further suppress conditions conducive to NO formation, offering another mechanistic explanation for reduced cumulative NO emissions observed in the manure + NI treatment.

### 4.2 CH_4_ and CO_2_ emissions

Cumulative CH_4_ emissions were generally low across all treatments and soils, with significant differences limited to the Plainfield Sand and Ringwood Silt Loam (Figure 6). Interestingly, in these soils the no‐manure treatment had significantly (*p* < 0.05) greater CH_4_ emissions than the manured soils despite receiving less C input. Greater cumulative CH_4_ emissions in the no‐manure treatment may reflect that methanogenesis occurred in some anaerobic microsites in the Plainfield Sand and Ringwood Silt Loam once NO_3_^−^ was depleted later in the incubation (Kim et al., 2015). Recent studies provide support for localized methanogenesis in anaerobic microsites despite macro‐level aerobic conditions (R. Angel et al., 2011; J. C. Angle et al., 2017; Lacroix et al., 2022). In manure‐amended soils, sustained N availability (as NH_4_^+^ and NO_3_^−^) and abundant DOC appeared to support ongoing denitrification (i.e., increased cumulative N_2_O and NO emissions), thereby inhibiting methanogenesis (i.e., decreased cumulative CH_4_ emissions) (Kim et al., 2015; Yuan & Lu, 2009). This is consistent with our hypothesis that methanogenesis would remain largely unchanged or suppressed under manure amendments, especially those that enhanced N cycling.

As hypothesized, manure amendments increased cumulative CO_2_ emissions compared to the no‐manure treatment (Figure 7). This likely reflects the substantial input of organic carbon (i.e., DOC) that stimulated microbial respiration, consistent with prior studies showing that labile C inputs from manure stimulate heterotrophic activity and early CO_2_ production (Jarecki et al., 2008; P. Li et al., 2017). Although the difference was only statistically significant (*p *< 0.05) in the Ringwood Silt Loam, soils receiving acidified manure tended to emit less CO_2_ than those receiving manure + NI (Figure 7). The relatively higher cumulative CO_2_ emissions in the manure + NI treatments may be attributed to DCD reducing nitrification‐driven H^+^ accumulation, thereby maintaining more favorable pH conditions for microbial respiration (Oertel et al., 2016).

## 5 CONCLUSION

This study shows that manure acidification and nitrification inhibition (DCD) influence GHG emissions and N cycling in soil‐specific ways. Acidified manure enhanced early NH_4_^+^ retention, which may reflect reduced NH_3_ volatilization under lower pH conditions, but shifted gaseous N losses toward N_2_O and NO, especially in coarse‐textured soils with low buffering capacity. These changes are consistent with pH‐mediated inhibition of nitrification and incomplete denitrification, where lower pH conditions could indirectly reduce CO_2_ emissions by suppressing heterotrophic respiration, though it was not directly measured. In contrast, DCD‐treated manure consistently delayed NH_4_^+^ oxidation and lowered NO and N_2_O emissions across all soils without reducing N mineralization or CO_2_ production, preserving plant‐available N. Methane emissions were generally low and unaffected by treatments but varied slightly among soils, indicating that inherent soil characteristics influenced occasional methanogenesis. These results indicate that NIs provide the greatest benefit for reducing N_2_O and NO losses, while manure acidification may reduce NH_3_ volatilization. Because these amendments act through partially overlapping mechanisms, combining them may yield additive benefits, particularly in soils with low buffering capacity. Overall, aligning manure management strategies with specific soil characteristics can optimize nutrient use efficiency and mitigate gas emissions, supporting more sustainable and climate‐resilient nutrient management in livestock‐based systems.

## AUTHOR CONTRIBUTIONS

**Mitch D. Wodrich**: Conceptualization; data curation; formal analysis; investigation; methodology; validation; visualization; writing—original draft. **Steven J. Hall**: Data curation; methodology; resources; writing—review and editing. **Xia Zhu‐Barker**: Conceptualization; funding acquisition; investigation; methodology; project administration; resources; supervision; validation; writing—review and editing.

## CONFLICT OF INTEREST STATEMENT

The authors declare conflicts of interest.

## Supporting information




**Supplemental Material**: Coefficient estimates from linear models describing the effects of treatment, soil type, and their interactions on cumulative mineralized N and cumulative nitrified N (Supplemental Table S1) and on cumulative N_2_O, NO, CH_4_, and CO_2_ emissions at Day 28 of the incubation period (Supplemental Table S5). Supplemental Table S2 presents coefficient estimates from linear models describing the effects of treatment, soil type, day range, and their interactions on net N mineralization rates and net nitrification rate. Supplemental Tables S3 and S4 present net N mineralization and nitrification rates, respectively, for each incubation period, along with cumulative mineralized N and cumulative nitrified NO_3_^−^ values over the entire incubation.
