# Supplementary material for: Acidification and nitrification inhibition of manure alters greenhouse gas emissions and nitrogen cycling in diverse agricultural soils
Source: J Environ Qual. 2025 Dec 14;55(1):e70123. doi: 10.1002/jeq2.70123 (PMC12703222; doi:10.1002/jeq2.70123)
Supplement: Supplementary file 1 — Supplemental Material: Coefficient estimates from linear models describing the effects of treatment, soil type, and their interactions on cumulative mineralized N and cumulative nitrified N (Supplemental Table S1) and on cumulative N2O, NO, CH4, and CO2 emissions at Day 28 of the incubation period (Supplemental Table S5). Supplemental Table S2 presents coefficient estimates from linear models describing the effects of treatment, soil type, day range, and their interactions on net N mineralization rates and net nitrification rate. Supplemental Tables S3 and S4 present net N mineralization and nitrification rates, respectively, for each incubation period, along with cumulative mineralized N and cumulative nitrified NO3 − values over the entire incubation. [file JEQ2-55-0-s001.docx]

**Acidification and Nitrification Inhibition of Manure Alters Greenhouse Gas Emissions and Nitrogen Cycling in Diverse Agricultural Soils**

Mitch D. Wodrich^1^, Steven J. Hall^2^, Xia Zhu-Barker^1*^

^1^ Department of Soil and Environmental Sciences, University of Wisconsin-Madison, Madison, WI 53706, USA

^2^ Department of Plant and Agroecosystem Sciences, University of Wisconsin-Madison, WI 53706, USA

^*^Corresponding author: [zhubarker@wisc.edu](mailto:zhubarker@wisc.edu)

**Supplemental Material**

**Pages**: 8

**Figures**: 0

**Tables**: 5

| **Table S1.** Coefficient estimates (B), standard errors, and p-values from linear models describing the effects of treatment, soil type, and their interaction on cumulative mineralized N and cumulative nitrified N (mg N kg^-1^ soil). Coefficients represent deviations from the reference level (Manure in Plano Silt Loam 1). | | | | | | | | |
| --- | --- | --- | --- | --- | --- | --- | --- | --- |
|  | | **Cumulative Mineralized N**  **(mg N kg^-1^ soil)** | | | | **Cumulative Nitrified N**  **(mg N kg^-1^ soil)** | | |
|  | **B** | | ***p*-value** | | **B** | | ***p*-value** | |
| **Intercept** | | 28.119 ±2.22 | | <0.001 | | 79.529 ±2.23 | | <0.001 |
| **Manure+Acid** | | -3.281 ±3.51 | | 0.36 | | -8.111 ±3.16 | | 0.017 |
| **Manure+NI** | | -2.640 ±3.14 | | 0.41 | | -77.252 ±3.16 | | <0.001 |
| **Plano Silt Loam 1** | | -6.795 ±3.14 | | 0.041 | | 11.594 ±3.54 | | 0.003 |
| **Plano Silt Loam 2** | | -16.914 ±3.14 | | <0.001 | | 1.376 ±3.16 | | 0.667 |
| **Ringwood Silt Loam** | | -18.922 ±3.14 | | <0.001 | | -4.889 ±3.16 | | 0.136 |
| **Manure+Acid × Plano Silt Loam 1** | | -4.225 ±4.71 | | 0.379 | | 11.877 ±4.75 | | 0.019 |
| **Manure+NI × Plano Silt Loam 1** | | 2.602 ±4.44 | | 0.564 | | 74.587 ±4.75 | | <0.001 |
| **Manure+Acid × Plano Silt Loam 2** | | 4.946 ±4.71 | | 0.305 | | 13.649 ±4.47 | | 0.005 |
| **Manure+NI × Plano Silt Loam 2** | | -0.505 ±4.44 | | 0.910 | | 64.630 ±4.47 | | <0.001 |
| **Manure+Acid × Ringwood Silt Loam** | | 7.255 ±4.71 | | 0.137 | | 6.326 ±4.47 | | 0.171 |
| **Manure+NI × Ringwood Silt Loam** | | 1.836 ±4.44 | | 0.683 | | 74.078 ±4.47 | | <0.001 |

| **Table S2.** Coefficient estimates (B), standard errors, and p-values from linear models describing the effects of treatment, soil type, day range, and their interaction on net N mineralization rate and net nitrification rate (mg N kg^-1^ d^-1^). Coefficients represent deviations from the reference level (Manure in Plano Silt Loam 1 between Days 0-1). | | | | | | |  |
| --- | --- | --- | --- | --- | --- | --- | --- |
|  | | **Net N Mineralization Rate**  **(mg N kg^-1^ d^-1^)** | | **Net Nitrification Rate**  **(mg N kg^-1^ d^-1^)** | | | |
|  | **B** | | ***p*-value** | **B** | ***p*-value** | | |
| **Intercept** | | -17.462 ±1.81 | <0.001 | 4.516 ±1.55 | | 0.004 |  |
| **Manure+NI** | | 10.774 ±2.57 | <0.001 | 0.597 ±2.2 | | 0.786 |  |
| **Manure+Acid** | | 6.398 ±2.57 | 0.013 | -1.147 ±2.2 | | 0.603 |  |
| **Plano Silt Loam 2** | | 0.080 ±2.57 | 0.974 | -2.626 ±2.2 | | 0.235 |  |
| **Plainfield Sand** | | -10.183 ±2.57 | <0.001 | -6.911 ±2.2 | | 0.002 |  |
| **Ringwood Silt Loam** | | -5.123 ±2.57 | 0.048 | -0.922 ±2.2 | | 0.676 |  |
| **Day Range 1-3** | | 21.413 ±2.57 | <0.001 | 11.218 ±2.2 | | <0.001 |  |
| **Day Range 3-5** | | 15.026 ±2.57 | <0.001 | 2.633 ±2.2 | | 0.234 |  |
| **Day Range 5-7** | | 19.482 ±2.57 | <0.001 | -1.726 ±2.2 | | 0.434 |  |
| **Day Range 7-14** | | 18.959 ±2.57 | <0.001 | -2.997 ±2.2 | | 0.175 |  |
| **Day Range 14-21** | | 17.264 ±2.57 | <0.001 | -4.709 ±2.2 | | 0.034 |  |
| **Day Range 21-28** | | 18.016 ±2.87 | <0.001 | -3.965 ±2.46 | | 0.109 |  |
| **Manure+NI × Plano Silt Loam 2** | | -8.107 ±3.63 | 0.027 | -1.660 ±3.11 | | 0.595 |  |
| **Manure+Acid × Plano Silt Loam 2** | | 4.425 ±3.63 | 0.225 | -1.901 ±3.11 | | 0.542 |  |
| **Manure+NI × Plainfield Sand** | | -10.590 ±3.63 | 0.004 | 0.261 ±3.11 | | 0.933 |  |
| **Manure+Acid × Plainfield Sand** | | 16.721 ±3.63 | <0.001 | 2.254 ±3.11 | | 0.470 |  |
| **Manure+NI × Ringwood Silt Loam** | | -10.040 ±3.63 | 0.006 | 1.352 ±3.11 | | 0.665 |  |
| **Manure+Acid × Ringwood Silt Loam** | | 2.924 ±3.63 | 0.422 | -1.155 ±3.11 | | 0.711 |  |
| **Manure+NI × Day Range 1-3** | | -22.020 ±3.63 | <0.001 | -13.751 ±3.11 | | <0.001 |  |
| **Manure+Acid × Day Range 1-3** | | -8.798 ±3.63 | 0.016 | -4.716 ±3.11 | | 0.132 |  |
| **Manure+NI × Day Range 3-5** | | -3.835 ±3.63 | 0.293 | 4.635 ±3.11 | | 0.139 |  |
| **Manure+Acid × Day Range 3-5** | | -9.032 ±3.63 | 0.014 | 0.199 ±3.11 | | 0.949 |  |
| **Manure+NI × Day Range 5-7** | | -6.623 ±3.63 | 0.070 | -1.505 ±3.11 | | 0.629 |  |
| **Manure+Acid × Day Range 5-7** | | -4.857 ±3.63 | 0.183 | 2.505 ±3.11 | | 0.422 |  |
| **Manure+NI × Day Range 7-14** | | -12.55 ±3.63 | <0.001 | 0.449 ±3.11 | | 0.885 |  |
| **Manure+Acid × Day Range 7-14** | | -6.492 ±3.63 | 0.076 | 2.442 ±3.11 | | 0.434 |  |
| **Manure+NI × Day Range 14-21** | | -11.553 ±3.63 | 0.001 | -0.744 ±3.11 | | 0.811 |  |
| **Manure+Acid × Day Range 14-21** | | -6.885 ±3.63 | 0.060 | 0.960 ±3.11 | | 0.758 |  |
| **Manure+NI × Day Range 21-28** | | -9.500 ±3.85 | 0.014 | 0.683 ±3.3 | | 0.836 |  |
| **Manure+Acid × Day Range 21-28** | | -5.141 ±3.85 | 0.184 | 2.422 ±3.3 | | 0.464 |  |
| **Plano Silt Loam 2 × Day Range 1-3** | | -14.168 ±3.63 | <0.001 | -17.967 ±3.11 | | <0.001 |  |
| **Plainfield Sand × Day Range 1-3** | | 9.043 ±3.63 | 0.013 | -8.083 ±3.11 | | 0.010 |  |
| **Ringwood Silt Loam × Day Range 1-3** | | -1.991 ±3.63 | 0.584 | -4.355 ±3.11 | | 0.164 |  |
| **Plano Silt Loam 2 × Day Range 3-5** | | 14.878 ±3.63 | <0.001 | 18.249 ±3.11 | | <0.001 |  |
| **Plainfield Sand × Day Range 3-5** | | 17.835 ±3.63 | <0.001 | 8.099 ±3.11 | | 0.010 |  |
| **Ringwood Silt Loam × Day Range 3-5** | | 20.483 ±3.63 | <0.001 | 9.242 ±3.11 | | 0.003 |  |
| **Plano Silt Loam 2 × Day Range 5-7** | | -2.169 ±3.63 | 0.551 | 4.319 ±3.11 | | 0.167 |  |
| **Plainfield Sand × Day Range 5-7** | | 12.206 ±3.63 | <0.001 | 13.137 ±3.11 | | <0.001 |  |
| **Ringwood Silt Loam × Day Range 5-7** | | -1.685 ±3.63 | 0.643 | -4.811 ±3.11 | | 0.124 |  |
| **Plano Silt Loam 2 × Day Range 7-14** | | -0.690 ±3.63 | 0.849 | 3.049 ±3.11 | | 0.329 |  |
| **Plainfield Sand × Day Range 7-14** | | 9.314 ±3.63 | 0.011 | 10.119 ±3.11 | | 0.001 |  |
| **Ringwood Silt Loam × Day Range 7-14** | | 4.667 ±3.63 | 0.201 | 0.463 ±3.11 | | 0.881 |  |
| **Plano Silt Loam 2 × Day Range 14-21** | | -1.326 ±3.63 | 0.715 | 1.429 ±3.11 | | 0.647 |  |
| **Plainfield Sand × Day Range 14-21** | | 10.757 ±3.63 | 0.003 | 7.546 ±3.11 | | 0.016 |  |
| **Ringwood Silt Loam × Day Range 14-21** | | 6.200 ±3.63 | 0.090 | 2.007 ±3.11 | | 0.520 |  |
| **Plano Silt Loam 2 × Day Range 21-28** | | 0.764 ±3.85 | 0.843 | 3.486 ±3.3 | | 0.293 |  |
| **Plainfield Sand × Day Range 21-28** | | 10.154 ±3.85 | 0.009 | 6.943 ±3.3 | | 0.037 |  |
| **Ringwood Silt Loam × Day Range 21-28** | | 4.476 ±3.85 | 0.247 | 0.161 ±3.3 | | 0.961 |  |
| **Manure+NI × Plano Silt Loam 2 × Day Range 1-3** | | 26.631 ±5.14 | <0.001 | 16.587 ±4.41 | | <0.001 |  |
| **Manure+Acid × Plano Silt Loam 2 × Day Range 1-3** | | 1.168 ±5.14 | 0.820 | 12.912 ±4.41 | | 0.003 |  |
| **Manure+NI × Plainfield Sand × Day Range 1-3** | | 24.699 ±5.14 | <0.001 | 10.886 ±4.41 | | 0.014 |  |
| **Manure+Acid × Plainfield Sand × Day Range 1-3** | | -23.367 ±5.14 | <0.001 | 1.494 ±4.41 | | 0.735 |  |
| **Manure+NI × Ringwood Silt Loam × Day Range 1-3** | | 25.892 ±5.14 | <0.001 | 2.406 ±4.41 | | 0.585 |  |
| **Manure+Acid × Ringwood Silt Loam × Day Range 1-3** | | 3.447 ±5.14 | 0.503 | 7.308 ±4.41 | | 0.099 |  |
| **Manure+NI × Plano Silt Loam 2 × Day Range 3-5** | | -4.823 ±5.14 | 0.349 | -17.731 ±4.41 | | <0.001 |  |
| **Manure+Acid × Plano Silt Loam 2 × Day Range 3-5** | | -9.083 ±5.14 | 0.079 | -7.443 ±4.41 | | 0.093 |  |
| **Manure+NI × Plainfield Sand × Day Range 3-5** | | -0.225 ±5.14 | 0.965 | -12.428 ±4.41 | | 0.005 |  |
| **Manure+Acid × Plainfield Sand × Day Range 3-5** | | -15.852 ±5.14 | 0.002 | -4.978 ±4.41 | | 0.260 |  |
| **Manure+NI × Ringwood Silt Loam × Day Range 3-5** | | -6.181 ±5.14 | 0.231 | -16.546 ±4.41 | | <0.001 |  |
| **Manure+Acid × Ringwood Silt Loam × Day Range 3-5** | | -14.671 ±5.14 | 0.004 | -0.613 ±4.41 | | 0.889 |  |
| **Manure+NI × Plano Silt Loam 2 × Day Range 5-7** | | 6.637 ±5.14 | 0.198 | 0.150 ±4.41 | | 0.972 |  |
| **Manure+Acid × Plano Silt Loam 2 × Day Range 5-7** | | -7.121 ±5.14 | 0.168 | 1.871 ±4.41 | | 0.671 |  |
| **Manure+NI × Plainfield Sand × Day Range 5-7** | | 8.655 ±5.14 | 0.094 | -9.193 ±4.41 | | 0.038 |  |
| **Manure+Acid × Plainfield Sand × Day Range 5-7** | | -17.595 ±5.14 | <0.001 | -7.857 ±4.41 | | 0.076 |  |
| **Manure+NI × Ringwood Silt Loam × Day Range 5-7** | | 11.23 ±5.14 | 0.030 | 2.889 ±4.41 | | 0.513 |  |
| **Manure+Acid × Ringwood Silt Loam × Day Range 5-7** | | 1.708 ±5.14 | 0.740 | 4.229 ±4.41 | | 0.338 |  |
| **Manure+NI × Plano Silt Loam 2 × Day Range 7-14** | | 10.197 ±5.14 | 0.049 | 0.407 ±4.41 | | 0.926 |  |
| **Manure+Acid × Plano Silt Loam 2 × Day Range 7-14** | | -3.592 ±5.14 | 0.485 | 2.521 ±4.41 | | 0.568 |  |
| **Manure+NI × Plainfield Sand × Day Range 7-14** | | 12.332 ±5.14 | 0.017 | -5.911 ±4.41 | | 0.181 |  |
| **Manure+Acid × Plainfield Sand × Day Range 7-14** | | -16.479 ±5.14 | 0.001 | -3.052 ±4.41 | | 0.489 |  |
| **Manure+NI × Ringwood Silt Loam × Day Range 7-14** | | 11.148 ±5.14 | 0.031 | -1.991 ±4.41 | | 0.652 |  |
| **Manure+Acid × Ringwood Silt Loam × Day Range 7-14** | | -3.618 ±5.14 | 0.482 | -0.846 ±4.41 | | 0.847 |  |
| **Manure+NI × Plano Silt Loam 2 × Day Range 14-21** | | 9.097 ±5.14 | 0.078 | 3.426 ±4.41 | | 0.438 |  |
| **Manure+Acid × Plano Silt Loam 2 × Day Range 14-21** | | -2.970 ±5.14 | 0.564 | 3.173 ±4.41 | | 0.472 |  |
| **Manure+NI × Plainfield Sand × Day Range 14-21** | | 11.106 ±5.14 | 0.032 | -0.448 ±4.41 | | 0.919 |  |
| **Manure+Acid × Plainfield Sand × Day Range 14-21** | | -16.683 ±5.14 | 0.001 | -1.277 ±4.41 | | 0.772 |  |
| **Manure+NI × Ringwood Silt Loam × Day Range 14-21** | | 10.831 ±5.14 | 0.036 | 1.310 ±4.41 | | 0.766 |  |
| **Manure+Acid × Ringwood Silt Loam × Day Range 14-21** | | -3.153 ±5.14 | 0.540 | 0.629 ±4.41 | | 0.886 |  |
| **Manure+NI × Plano Silt Loam 2 × Day Range 21-28** | | 5.860 ±5.3 | 0.270 | 1.546 ±4.54 | | 0.734 |  |
| **Manure+Acid × Plano Silt Loam 2 × Day Range 21-28** | | -6.360 ±5.3 | 0.232 | -0.058 ±4.54 | | 0.989 |  |
| **Manure+NI × Plainfield Sand × Day Range 21-28** | | 8.555 ±5.3 | 0.108 | -2.395 ±4.54 | | 0.598 |  |
| **Manure+Acid × Plainfield Sand × Day Range 21-28** | | -18.320 ±5.3 | <0.001 | -3.267 ±4.54 | | 0.473 |  |
| **Manure+NI × Ringwood Silt Loam × Day Range 21-28** | | 8.443 ±5.3 | 0.113 | -1.711 ±4.54 | | 0.707 |  |
| **Manure+Acid × Ringwood Silt Loam × Day Range 21-28** | | -3.365 ±5.3 | 0.526 | 0.8 ±4.54 | | 0.860 |  |

| **Table S3.** Net N mineralization rates for each incubation period and cumulative mineralized N over the entire incubation course. Uppercase letters indicate significant differences among soil types within each treatment and incubation period. Lowercase letters indicate significant differences among treatments within each soil type and incubation period. Greek letters indicate significant differences among incubation periods within each soil type and treatment. Significance was determined at α = 0.05 (n = 3). | | | | | | |
| --- | --- | --- | --- | --- | --- | --- |
| **Net N Mineralization Rate (mg N kg^-1^ d^-1^)** | | | | | | |
| **Period (d)** | **Manure** | | **Manure + NI** | | **Manure + Acid** | |
| **Plano Silt Loam 1** |  |  |  |  |  |  |
| 0-1 | -17.46 | ±0.78 Abβ | -6.69 | ±7.73 Aaβ | -11.06 | ±0.70 ABaγ |
| 1-3 | 3.95 | ±3.15 Aaα | -7.29 | ±4.47 Cbβ | -7.29 | ±4.47 Aaαβ |
| 3-5 | -2.44 | ±3.96 Cbα | 4.5 | ±6.18 Aaα | 4.5 | ±6.18 Cbβγ |
| 5-7 | 2.02 | ±0.68 Aaα | 6.17 | ±1.26 Aaα | 6.17 | ±1.26 Aaα |
| 7-14 | 1.5 | ±0.36 Aaα | -0.28 | ±0.49 Aaαβ | -0.28 | ±0.49 Aaαβ |
| 14-21 | -0.2 | ±0.54 Aaα | -0.98 | ±0.94 Aaαβ | -0.98 | ±0.94 Aaαβ |
| 21-28 | 0.55 | ±0.84 Aaα | 1.83 | ±1.53 Aaα | 1.83 | ±1.53 Aaαβ |
| Cumulative Mineralized N (mg N kg^-1^) | 24.84 | ±4.10 Aa | 25.48 | ±3.91 Aa | 25.48 | ±3.91 Aa |
| **Plano Silt Loam 2** |  |  |  |  |  |  |
| 0-1 | -17.38 | ±1.64 Abγ | -14.71 | ±1.26 Bb**γ** | -14.71 | ±1.26 Aaβ |
| 1-3 | -10.14 | ±2.69 Cbγ | -2.86 | ±1.59 BCaβ | -2.86 | ±1.59 Babβ |
| 3-5 | 12.52 | ±4.44 Aaα | 6.53 | ±1.40 Aabα | 6.53 | ±1.40 Abα |
| 5-7 | -0.07 | ±1.32 ABaβ | 2.61 | ±1.52 Aaαβ | 2.61 | ±1.52 Aaαβ |
| 7-14 | 0.89 | ±0.84 Aaβ | 1.2 | ±0.28 Aaαβ | 1.2 | ±0.28 Aaα |
| 14-21 | -1.44 | ±2.02 Aaβ | -1.23 | ±0.75 Aaβ | -1.23 | ±0.75 Aaαβ |
| 21-28 | 1.4 | ±2.19 Aaβ | 0.43 | ±0.48 Aaαβ | 0.43 | ±0.48 Aaαβ |
| Cumulative Mineralized N (mg N kg^-1^) | 13.82 | ±1.06 Ba | 21.29 | ±1.45 Aa | 21.29 | ±1.45 Aa |
| **Plainfield Sand** |  |  |  |  |  |  |
| 0-1 | -27.65 | ±1.33 Bbβ | -27.46 | ±0.77 Cbβ | -27.46 | ±0.77 Aaβ |
| 1-3 | 2.81 | ±1.09 ABaα | 5.67 | ±0.82 Aaα | 5.67 | ±0.82 Bbβ |
| 3-5 | 5.22 | ±2.17 Baα | 1.34 | ±0.23 Aaα | 1.34 | ±0.23 ABaα |
| 5-7 | 4.04 | ±1.56 Aaα | 6.26 | ±0.06 Aaα | 6.26 | ±0.06 Aaα |
| 7-14 | 0.63 | ±0.61 Aaα | 0.6 | ±0.09 Aaα | 0.6 | ±0.09 Aaαβ |
| 14-21 | 0.38 | ±0.32 Aaα | 0.11 | ±0.13 Aaα | 0.11 | ±0.13 Aaαβ |
| 21-28 | 0.53 | ±0.29 Aaα | -0.24 | ±0.11 Aaα | -0.24 | ±0.11 Aaαβ |
| Cumulative Mineralized N (mg N kg^-1^) | 12.87 | ±1.33 Ba | 8.06 | ±0.64 Ba | 8.06 | ±0.64 Ba |
| **Ringwood Silt Loam** |  |  |  |  |  |  |
| 0-1 | -22.59 | ±1.19 Bαβγ | -21.85 | ±1.10 Cbβ | -21.85 | ±1.10 Baβ |
| 1-3 | -3.16 | ±1.11 Baβ | 1.44 | ±0.25 ABaα | 1.44 | ±0.25 Aaα |
| 3-5 | 12.92 | ±1.40 Aaα | 3.64 | ±0.98 Abα | 3.64 | ±0.98 BCbα |
| 5-7 | -4.79 | ±0.31 Bbβ | 0.55 | ±1.35 Aabα | 0.55 | ±1.35 Aaα |
| 7-14 | 1.04 | ±0.26 Aaβ | 0.37 | ±0.25 Aaα | 0.37 | ±0.25 Aaα |
| 14-21 | 0.88 | ±0.34 Aaβ | 0.89 | ±0.43 Aaα | 0.89 | ±0.43 Aaα |
| 21-28 | -0.09 | ±0.29 Aaβ | -0.42 | ±0.48 Aaα | -0.42 | ±0.48 Aaα |
| Cumulative Mineralized N (mg N kg^-1^) | 13.17 | ±1.45 Ba | 8.39 | ±2.35 Ba | 8.39 | ±2.35 Ba |

| **Table S4.** Net nitrification rates for each incubation period and cumulative nitrified NO_3_^-^ over the entire incubation course. Uppercase letters indicate significant differences among soil types within each treatment and incubation period. Lowercase letters indicate significant differences among treatments within each soil type and incubation period. Greek letters indicate significant differences among incubation periods within each soil type and treatment. Significance was determined at α = 0.05 (n = 3). | | | | | | |
| --- | --- | --- | --- | --- | --- | --- |
| **Net Nitrification Rate (mg N kg^-1^ d^-1^)** | | | | | | |
| **Period (d)** | **Manure** | | **Manure + NI** | | **Manure + Acid** | |
| **Plano Silt Loam 1** |  |  |  |  |  |  |
| 0-1 | 4.52 | ±0.70 Aaβγ | 5.11 | ±1.63 Aaβ | 3.37 | ±0.55 Aaαβγ |
| 1-3 | 15.74 | ±2.91 Aaα | 2.58 | ±5.96 Acβ | 9.87 | ±1.44 Abα |
| 3-5 | 7.15 | ±2.93 Cbβ | 12.38 | ±5.65 Aaα | 6.2 | ±2.34 Bbαβ |
| 5-7 | 2.79 | ±1.28 Baβγ | 1.88 | ±1.34 Aaβ | 4.15 | ±0.90 Aaαβγ |
| 7-14 | 1.52 | ±0.36 Aaβγ | 2.57 | ±0.03 Aaβ | 2.81 | ±0.38 Aaβγ |
| 14-21 | -0.19 | ±0.54 Aaγ | -0.34 | ±0.81 Aaβ | -0.38 | ±0.83 Aaγ |
| 21-28 | 0.55 | ±0.85 Aaβγ | 1.83 | ±1.53 Aaβ | 1.83 | ±1.04 Aaβγ |
| Cumulative Nitrified NO_3_^-^ (mg N kg^-1^) | 91.12 | ±4.12 Aa | 88.46 | ±3.92 Aa | 94.89 | ±3.03 Aa |
| **Plano Silt Loam 2** |  |  |  |  |  |  |
| 0-1 | 1.89 | ±1.48 ABaβ | 0.83 | ±1.00 Aaβ | -1.16 | ±0.44 Aaγ |
| 1-3 | -4.86 | ±2.51 Baγ | -3.08 | ±0.29 Aaβ | 0.29 | ±1.31 Baβγ |
| 3-5 | 22.77 | ±4.39 Aaα | 8.61 | ±0.65 ABbα | 12.48 | ±2.02 Abα |
| 5-7 | 4.48 | ±1.43 ABaβ | 2.06 | ±0.53 Aaαβ | 5.81 | ±2.90 Aaβ |
| 7-14 | 1.94 | ±0.80 Aaβ | 1.74 | ±0.14 Aaβ | 3.86 | ±0.75 Aaβγ |
| 14-21 | -1.39 | ±2.01 Aaβγ | 0.23 | ±1.14 Aaβ | -0.3 | ±0.08 Aaβγ |
| 21-28 | 1.41 | ±2.19 Aaβγ | 2.58 | ±1.13 Aaαβ | 0.73 | ±0.24 Aaβγ |
| Cumulative Nitrified NO_3_^-^ (mg N kg^-1^) | 80.91 | ±1.06 Ba | 68.28 | ±1.00 Bb | 86.44 | ±2.83 Aa |
| **Plainfield Sand** |  |  |  |  |  |  |
| 0-1 | -2.39 | ±0.33 Baγ | -1.54 | ±1.20 Baα | -1.29 | ±0.61 Aaαβ |
| 1-3 | 0.74 | ±0.88 Baβγ | -1.27 | ±0.88 Aaα | -1.37 | ±0.76 Baβ |
| 3-5 | 8.34 | ±1.65 Caα | 1.4 | ±1.23 Cbα | 4.67 | ±0.88 Babαβ |
| 5-7 | 9.02 | ±1.38 Aaα | -0.82 | ±0.40 Abα | 4.77 | ±0.50 Aaαβ |
| 7-14 | 4.73 | ±0.45 Aaαβ | 0.12 | ±0.07 Aaα | 5.22 | ±0.07 Aaα |
| 14-21 | 0.44 | ±0.29 Aaβγ | 0.11 | ±0.08 Aaα | 1.23 | ±0.44 Aaαβ |
| 21-28 | 0.58 | ±0.32 Aaβγ | -0.27 | ±0.16 Aaα | 0.84 | ±0.37 Aaαβ |
| Cumulative Nitrified NO_3_^-^ (mg N kg^-1^) | 79.53 | ±1.33 Ba | 2.28 | ±0.64 Cc | 71.42 | ±2.01 Bb |
| **Ringwood Silt Loam** |  |  |  |  |  |  |
| 0-1 | 3.59 | ±1.93 Aaβ | 5.54 | ±0.69 Aaα | 1.29 | ±1.52 Baα |
| 1-3 | 10.46 | ±0.28 Aaα | 1.06 | ±0.58 Abα | 10.75 | ±0.20 Aaα |
| 3-5 | 15.47 | ±2.12 Baα | 5.51 | ±0.62 BCbα | 12.75 | ±2.32 Aaα |
| 5-7 | -2.94 | ±1.66 Caβ | 0.39 | ±0.59 Aaα | 1.49 | ±1.88 Aaβ |
| 7-14 | 1.06 | ±0.26 Aaβ | 1.47 | ±0.24 Aaα | 0.35 | ±0.36 Aaβ |
| 14-21 | 0.89 | ±0.34 Aaβ | 3.41 | ±0.41 Aaα | 0.18 | ±0.80 Aaβ |
| 21-28 | -0.21 | ±0.17 Aaβ | 0.71 | ±0.18 Aaα | 0.71 | ±1.30 Aaβ |
| Cumulative Nitrified NO_3_^-^ (mg N kg^-1^) | 74.64 | ±0.83 Ba | 71.47 | ±2.43 Ba | 72.85 | ±1.31 Ba |

| **Table S5.** Coefficient estimates (B), standard errors, and p-values from linear models describing the effects of treatment, soil type, and their interaction on cumulative N_2_O, NO, CH_4_, and CO_2_ emissions at Day 28. Models were fit to transformed responses where appropriate (log-transformed for N_2_O, square-root–transformed for NO, and untransformed for CH_4_ and CO_2_). Coefficients for N_2_O are unitless and represent effects on the log scale, coefficients for NO are expressed in √mg, and coefficients for CH4 and CO2 are expressed in mg and g, respectively. Coefficients represent deviations from the reference level (No Manure in Plano Silt Loam 1). | | | | | | | | | | | | |  |
| --- | --- | --- | --- | --- | --- | --- | --- | --- | --- | --- | --- | --- | --- |
|  | **N_2_O (Unitless)** | | **NO (√mg N)** | | | **CH_4_ (mg C)** | | | **CO_2_ (g C)** | | | | |
|  | **B** | ***p*-value** | | **B** | ***p*-value** | | **B** | ***p*-value** | | **B** | ***p*-value** |  |  |
| **Intercept** | 0.025  ±0.17 | 0.882 | | 0.159  ±0.15 | 0.293 | | -0.854  ±0.39 | 0.034 | | 6.271  ±1.54 | <0.001 |  |  |
| **Manure** | -0.495  ±0.24 | 0.048 | | 0.576  ±0.21 | 0.010 | | 0.550  ±0.54 | 0.320 | | 4.743  ±2.18 | 0.037 |  |  |
| **Manure+NI** | -0.898  ±0.24 | 0.001 | | 0.299  ±0.21 | 0.164 | | 0.314  ±0.54 | 0.568 | | 5.678  ±2.18 | 0.014 |  |  |
| **Manure+Acid** | -0.614  ±0.24 | 0.016 | | 0.815  ±0.21 | 0.000 | | 1.207  ±0.54 | 0.034 | | 0.072  ±2.18 | 0.974 |  |  |
| **Plano Silt Loam 2** | -1.217  ±0.24 | <0.001 | | 0.133  ±0.21 | 0.530 | | 1.571  ±0.54 | 0.007 | | -4.602  ±2.18 | 0.042 |  |  |
| **Plainfield Sand** | -1.203  ±0.24 | <0.001 | | 0.075  ±0.21 | 0.722 | | 4.080  ±0.54 | <0.001 | | -6.198  ±2.18 | 0.008 |  |  |
| **Ringwood Silt Loam** | -1.235  ±0.24 | <0.001 | | 0.225  ±0.21 | 0.292 | | 3.315  ±0.54 | <0.001 | | -2.600  ±2.18 | 0.241 |  |  |
| **Manure × Plano Silt Loam 2** | 1.354  ±0.34 | <0.001 | | -0.530  ±0.30 | 0.084 | | 0.036  ±0.77 | 0.963 | | 3.344  ±3.08 | 0.286 |  |  |
| **Manure+NI × Plano Silt Loam 2** | 1.148  ±0.34 | 0.002 | | -0.248  ±0.30 | 0.410 | | 0.373  ±0.77 | 0.631 | | 3.177  ±3.08 | 0.310 |  |  |
| **Manure+Acid × Plano Silt Loam 2** | 1.279  ±0.34 | <0.001 | | -0.537  ±0.30 | 0.080 | | -0.221  ±0.77 | 0.775 | | 6.119  ±3.08 | 0.056 |  |  |
| **Manure × Plainfield Sand** | 3.607  ±0.34 | <0.001 | | -0.273  ±0.30 | 0.365 | | -2.821  ±0.77 | 0.001 | | 6.178  ±3.08 | 0.053 |  |  |
| **Manure+NI × Plainfield Sand** | 1.236  ±0.34 | <0.001 | | -0.320  ±0.30 | 0.289 | | -1.825  ±0.77 | 0.024 | | 10.333  ±3.08 | 0.002 |  |  |
| **Manure+Acid × Plainfield Sand** | 4.639  ±0.34 | <0.001 | | -0.480  ±0.30 | 0.116 | | -3.484  ±0.77 | <0.001 | | 13.072  ±3.08 | <0.001 |  |  |
| **Manure × Ringwood Silt Loam** | 3.041  ±0.34 | <0.001 | | 0.007  ±0.30 | 0.980 | | -2.974  ±0.77 | 0.001 | | 3.240  ±3.08 | 0.301 |  |  |
| **Manure+NI × Ringwood Silt Loam** | 1.854  ±0.34 | <0.001 | | -0.259  ±0.30 | 0.390 | | -1.820  ±0.77 | 0.024 | | 4.787  ±3.08 | 0.130 |  |  |
| **Manure+Acid × Ringwood Silt Loam** | 3.284  ±0.34 | <0.001 | | -0.427  ±0.30 | 0.160 | | -1.871  ±0.77 | 0.021 | | 4.45  ±3.08 | 0.158 |  |  |
